# The structural and functional roles of the flavin cofactor FAD in mammalian cryptochromes

**DOI:** 10.3389/fmolb.2022.1081661

**Published:** 2023-01-04

**Authors:** Giulia Calloni, R. Martin Vabulas

**Affiliations:** ^1^ AB SCIEX Germany GmbH, Darmstadt, Germany; ^2^ Institute of Biochemistry, Charité-Universitätsmedizin Berlin, Berlin, Germany

**Keywords:** flavin cofactor, FAD, flavoprotein, cryptochrome, circadian rhythm

## Abstract

The importance of circadian rhythms in human health and disease calls for a thorough understanding of the underlying molecular machinery, including its key components, the flavin adenine dinucleotide (FAD)-containing flavoproteins cryptochrome 1 and 2. Contrary to their *Drosophila* counterparts, mammalian cryptochromes are direct suppressors of circadian transcription and act independently of light. Light-independence poses the question regarding the role of the cofactor FAD in mammalian cryptochromes. The weak binding of the cofactor *in vitro* argues against its relevance and might be a functionless evolutionary remnant. From the other side, the FAD-binding pocket constitutes the part of mammalian cryptochromes directly related to their ubiquitylation by the ubiquitin ligase Fbxl3 and is the target for protein-stabilizing small molecules. Increased supplies of FAD stabilize cryptochromes in cell culture, and the depletion of the FAD precursor riboflavin with simultaneous knock-down of riboflavin kinase affects the expression of circadian genes in mice. This review presents the classical and more recent studies in the field, which help to comprehend the role of FAD for the stability and function of mammalian cryptochromes.

## 1 Introduction

The Latin expression *circa diem* translates “about a day” and stands for the 24-h periodicity. Circadian rhythms entail diurnal and nocturnal variants, which refer to organisms active during daylight or at night, respectively. In constant darkness, both diurnal and nocturnal animals maintain 24-h periodicity in physiology and behavior, which points to the endogenous mechanisms of rhythmicity (Allada and Bass, 2021). Disruption of circadian rhythms have been associated with several pathologies and there are considerable efforts underway to exploit circadian mechanisms for diagnosis, treatment, and prevention of diseases (Kramer et al., 2022).

Circadian rhythms are driven by intracellular molecular clocks that operate as transcription-translation feedback loops. The protein components of these loops cooperate to generate cyclic changes in their own abundance and activity (Patke et al., 2020). In humans, the main clock entails the heterodimeric transcription factor Clock/Bmal1 and its inhibitors period (Per1, Per2, Per3) and cryptochrome (Cry1, Cry2). Clock/Bmal1 binds to E-box-containing gene regulatory sequences to drive transcription of circadian genes, including those of its own inhibitors Per and Cry. The inhibitors translocate into the nucleus and form a 1.9-MDa repressor assembly with Clock/Bmal1 to suppress the transcription (Aryal et al., 2017). In addition to the main clock, mammals have additional protein components that contribute to circadian cycling, for example, the nuclear receptor Rev-Erb and the retinoic acid-related orphan receptor α (RORα), which compete for binding to the ROR response elements in promoters and enhancers of target genes (Preitner et al., 2002; Sato et al., 2004).

## 2 Cryptochromes can act as flavin-dependent photoreceptors

Cryptochromes are the main transcriptional repressors of Clock/Bmal1. They constitute one of the three classes of blue light-sensing flavoproteins and are found in bacteria, fungi, plants and animals. Another class is the light-oxygen-voltage (LOV) domains typically found in fungi and plants (Krauss et al., 2009). Most recently discovered are the blue light sensing using FAD (BLUF) domains primarily found in bacteria (Gomelsky and Klug, 2002). Generally speaking, light induces chemical changes of flavin cofactors in these photoreceptors, that in turn lead to conformational rearrangement of proteins, which is responsible for the downstream effects upon light stimulation. There has been much progress in the mechanistic understanding of the light sensing by photoreceptors, including that by flavoproteins (Kottke et al., 2018).

It is commonly accepted that cryptochromes are evolutionary related to DNA photolyases (Miles et al., 2020; Deppisch et al., 2022). Both protein groups share structural homology and thus are usually considered as one cryptochrome/photolyase family. DNA-repairing photolyases transfer light energy to the fully reduced flavin cofactor which then donates electrons to break cross-linked pyrimidine dimers (Kao et al., 2005). Cryptochromes show very distinct functionalities, most famously operating in the circadian clock machinery in plants and animals. Initially, they were classified into three groups: CRY-DASH, plant cryptochromes, and animal cryptochromes (Lin and Todo, 2005). DASH stands for *Drosophila*, *Arabidopsis*, *Synechocystis*, Human to indicate that the *Arabidopsis* and *Synechocystis* photolyases of the branch are more related to *Drosophila* and human cryptochromes than to bacterial photolyases (Brudler et al., 2003). Yet, CRY-DASH members do show DNA repair activity (Selby and Sancar, 2006; Tagua et al., 2015; Navarro et al., 2020). The evolutionary relationship between different types of cryptochromes and photolyases has been recently reanalyzed (Ozturk, 2017, 2022).

All cryptochromes feature the conserved photolyase homology region (PHR) and the C-terminal extension (CCT). The CCT varies between different cryptochromes in its sequence and length and contributes to the initiation of cellular responses following excitation of FAD (Yang et al., 2000). Oxidized FAD bound to PHR represents the resting cryptochrome state. Upon light stimulation, electron transfer from tryptophan to flavin occurs (Kottke et al., 2018). The reduction of flavin results in an anionic semiquinone. Alternatively, if protonation from an adjacent amino acid takes place simultaneously, the reduction produces a neutral semiquinone flavin. Flavin reduction leads to conformational changes, release of the CCT and other rearrangements of the cryptochrome structure cumulatively ushering downstream effects of the light stimulus (Ozturk et al., 2014; Ganguly et al., 2016; Berntsson et al., 2019). Computational modelling predicts an Arg-Asp salt bridge acting as an allosteric switch that in turn is regulated by the redox state of FAD (Wang et al., 2021).

## 3 Type II animal cytochromes work light-independently

Animal cryptochromes can be subdivided in two types according to their light dependence. Type I are light-responsive cryptochromes, as exemplified by the *Drosophila* Cry (dCry). Contrary to mammals, dCry is not a part of the circadian transcription inhibitory complex. Tim/Per dimer is responsible for the inhibition in fruit flies. Light-induced changes enable dCry to interact with Tim and mediate its ubiquitylation by the ubiquitin ligase Jet (Koh et al., 2006). Without Tim, Per becomes a target for the ubiquitin ligase Slimb and is degraded as well (Grima et al., 2002; Hw et al., 2002). The loss of Tim and Per starts a new circadian cycle. Noteworthy, degradation of Tim exposes dCry and leads to its ubiquitylation and degradation (Peschel et al., 2009). Different affinities of Tim and dCry to the Jet ligase was proposed to underlie their sequential degradation.

Type II are light-nonresponsive cryptochromes, such as human Cry1 and Cry2. All analyzed vertebrates, among others zebrafish, clawed frog, and chicken, possess Type II cryptochromes. (Chaves et al., 2011b). In contrast to *Drosophila*-type proteins, type II chromophores act as direct and light-independent repressors of the circadian transcription complex (Griffin et al., 1999). Surprisingly, mice lacking Cry1 or Cry2 showed accelerated and delayed free-running periodicity of locomotor activity, respectively (van der Horst et al., 1999). One possible explanation of this dichotomy is the competition of the stronger suppressor Cry1 and the weaker suppressor Cry2 for a rate-limiting interaction with Per, which then determines the nuclear translocation and inhibitory activity of Per (Hirota et al., 2012). The individual knockouts obviously lack this competition. On the other hand, the double knock-out manifested immediate and complete loss of circadian rhythmicity when placed in constant darkness, which proved the central role of cryptochromes in the mammalian circadian machinery. Unexpectedly, the double-mutant mice retained the ability to induce the expression of Per1 and Per2 upon a brief light stimulus (Okamura et al., 1999). The opsin-like protein melanopsin in retinal ganglion cells was later proposed as the circadian photoreceptor responsible for the above effect (Lucas et al., 2003; Semo et al., 2003).

It was shown that mammalian Cry1 can inhibit Clock/Bmal1-driven transcription without dissociating the complex from DNA (“blocking”-type repression) (Ye et al., 2014). Per2 alone had no effect, however, in the presence of cryptochrome, Per entered the nucleus and was able to displace Clock/Bmal1 from the promoter (“displacement”-type repression). Crystal structures showed how Cry docks on Clock and suggested that Per can influence the binding of Cry to the transcription complex (Nangle et al., 2014; Michael et al., 2017). A further study provide functional evidence that Per can indeed enhance interaction of cryptochrome with Clock/Bmal1 (Rosensweig et al., 2018).

## 4 Mammalian cryptochromes associate with FAD very weakly

During their biogenesis, flavoproteins need to incorporate flavin cofactors to acquire the final structural and functional maturity. Mammals cannot synthesize riboflavin, the precursor of flavin mononucleotide (FMN) and flavin adenine dinucleotide (FAD) cofactors, and thus rely on its uptake from external sources. Three mammalian transporters of riboflavin have been identified, Slc52A1-3 (Yonezawa and Inui, 2013). Accordingly, FAD must be deadenylated to FMN and FMN further dephosphorylated to riboflavin, before cellular uptake of riboflavin can take place. Intracellularly, riboflavin is then transformed back to FMN and FAD by the riboflavin kinase (Rfk) and the flavin adenine dinucleotide synthetase (Flad1), respectively.

The association of flavin cofactors to apoenzymes *in vivo* is still insufficiently understood. Most mammalian cells do not have free flavin reserves, which means that flavoprotein synthesis and maturation must be coordinated with the supplies of the flavin cofactors. It is generally assumed that the cofactors bind to apoproteins in a non-assisted manner and that the process is driven solely by the high affinity of the interaction. Yet, there are indications that the association of flavin cofactors with apoproteins might be more complicated. For example, the affinity of Flad1 to its product FAD is very high, which suggests that Flad1 might function as an FAD chaperone that can only directly deliver FAD to the client flavoproteins (Torchetti et al., 2011; Giancaspero et al., 2015). Furthermore, the extent of cofactor-enzyme association may change depending on the cellular status or interaction partners present in flavoprotein complexes (Hashida et al., 2004; Yazdanpanah et al., 2009). More research is needed to understand the dynamics of flavin associations (Schnerwitzki and Vabulas, 2022).

The FAD-binding pocked of cryptochromes lies in the conserved photolyase homology region (PHR). Because of the similarity of the domain, it might appear surprising that animal Type I cryptochromes can be purified with stoichiometric amounts of FAD, while Type II (mammalian) cryptochromes isolated from different sources contain only residual amounts of the cofactor (Oztürk et al., 2007; Kutta et al., 2017). It is tempting to correlate the absence of the light-dependent function of mammalian cryptochromes with their weak association with FAD. The association constant (K_d_) was measured to be 16 μM and 68 μM for human Cry1 and Cry2, respectively (Kutta et al., 2017). Intracellular flavin concentrations vary somewhat between different cells, yet remain in the lower micromolar range (Hühner et al., 2015). In view of these levels, the determined K_d_’s imply only partial saturation of human cryptochromes with FAD in the cell. From the other side, local subcellular concentrations of metabolites can differ significantly from those measured in bulk. For example, a dynamic pool of FAD in the nucleus was described (Giancaspero et al., 2013). Because of the weak affinity to cryptochromes, changes of FAD concentration might be relevant for their stability and function.

## 5 Fbxl3 mediates degradation of cryptochromes

The mechanism of cellular degradation of mammalian cryptochromes was discovered in two independent genetic screens in mice (Godinho et al., 2007; Siepka et al., 2007). Both used the N-ethyl-N-nitrosurea (ENU) mutagenesis aiming to identify molecular determinants of circadian wheel-running activity. In one study the respective mutant was named “after hours” (*Afh*) (Godinho et al., 2007). A single locus of *Afh* linkage had been found on mouse chromosome 14, which was further refined to a gene-poor region containing only 25 annotated genes. Scanning and sequencing of the candidates detected a point mutation in an F-box gene, Fbxl3. The mutation results in the substitution C358S. A second study identified another mutant and named it “overtime” (*Ovtm*) (Siepka et al., 2007). Also *Ovtm* turned out to be a non-synonymous mutation in Fbxl3, this time resulting in the protein variant I364T. The isoleucine 364 is highly conserved in vertebrate Fbxl3 and in mouse paralogue Fbxl21.

Fbxl3 belongs to the family of so-called F-box proteins that owe their name to the eponymous domain originally found in the cyclin F (Bai et al., 1996). Fbxl3 is a member of the F-box protein subfamily characterized by the presence of leucine reach repeats which are often involved in protein-protein interactions (Jin et al., 2004). As one of four subunits in the E3 ubiquitin ligase complex SCF (Skp1-Cul1-F-box protein), F-box proteins are responsible for substrate recognition. Until now, around 70 F-box proteins have been identified in mammals. The E3 ligase complex containing Fbxl3 is called SCF^Fbxl3^ and was found to recognize and ubiquitylate Cry1 and Cry2 (Busino et al., 2007; Siepka et al., 2007). The interaction between Fbxl3 and cryptochromes was discovered by means of mass spectrometry analysis of the pulldowns of the overexpressed Fbxl3 in HeLa cells and was verified biochemically (Busino et al., 2007). The specificity of binding was tested using nine other F-box proteins and six other proteins from the cellular clock machinery, including Per1 and Per2. Importantly, the *Afh* variant of Fbxl3 failed to interact with Cry1 and showed reduced interaction with and *in vitro* ubiquitylation of Cry2. Similarly, the *Ovtm* variant was compared to wild-type Fbxl3 (Siepka et al., 2007). In this latter setup, the interaction of the variant with cryptochromes was comparable, however, the variant induced less degradation of Cry1 compared to the wild-type Fbxl3. Of note, the stability of *Ovtm* itself seemed to be affected by the mutation. Thus, both the decreased capacity of *Ovtm* to ubiquitylate cryptochromes and the lower availability of the mutant ubiquitin ligase contribute to the observed circadian disturbances in the mutant mice.

## 6 Fbxl21 mediates degradation of cryptochromes in the cytosol

The complexity of cellular degradation of cryptochromes was uncovered by two further genetic studies that identified an additional ubiquitin ligase, Fbxl21, which interferes with the Fbxl3-driven degradation of Cry in the nucleus. One study was another ENU screen in mice (Yoo et al., 2013). The authors isolated and cloned the circadian mutant “past-time” (*Psttm*) which shortens circadian period and destabilizes cryptochromes during the circadian cycle. The linkage locus contained 167 open reading frames with no known circadian clock genes. Yet, it contained Fbxl21, a paralogue of Fbxl3, that turned out to bear the G149E mutation, which creates a charged protrusion and thus is expected to destabilize the LRR domain of the protein. When compared to Fbxl3, Fbxl21 interacted with Cry more strongly, at the same time it was less efficient in the ubiquitylation activity. One of the reasons for this apparent discrepancy might be the number of lysine targets of the different ubiquitin ligases: many lysine residues in the case of Fbxl3 vs. only one preferred lysine (K11) in the case of Fbxl21. Also the second study documented weaker ubiquitylation of cryptochromes by Fbxl21 (Hirano et al., 2013). Here, the authors prepared and compared Fbxl3 and Fbxl21 knock-out mice. The antagonistic interplay of both ligases became evident in the double knock-out: the combined deficiency showed alleviation of the circadian period-lengthening phenotype seen in the single knock-out of Fbxl3. Furthermore, both studies revealed preferentially different localization of the ligases in the cell: Fbxl3 was found in the nucleus while Fbxl21 acted mainly in the cytosol. Strong binding but weak ubiquitylation of Cry by Fbxl21 in the cytosol can be seen as a form of sequestration and protection of Cry from its strong ubiquitylation by Fbxl3 in the nucleus.

## 7 Ubiquitylation of cryptochromes requires FAD-free proteins

In plant and insect cryptochromes and related photolyases, FAD cofactor is buried deep inside the cofactor pocket in crystal structures. The structure of murine FAD-bound cryptochrome 2 came as a surprise, because it revealed quite a different arrangement (Xing et al., 2013). Noteworthy, the structure was determined by reconstituting purified apo-Cry with high concentrations of FAD. The cofactor was only partially embedded in murine Cry2 and exposed one side of its adenosine diphosphate to the solvent. The exposed conformation fits well with the low apparent FAD dissociation constant and explains the loss of the cofactor while purifying mammalian cryptochromes from endogenous sources or upon heterologous expression.

**FIGURE 1:**
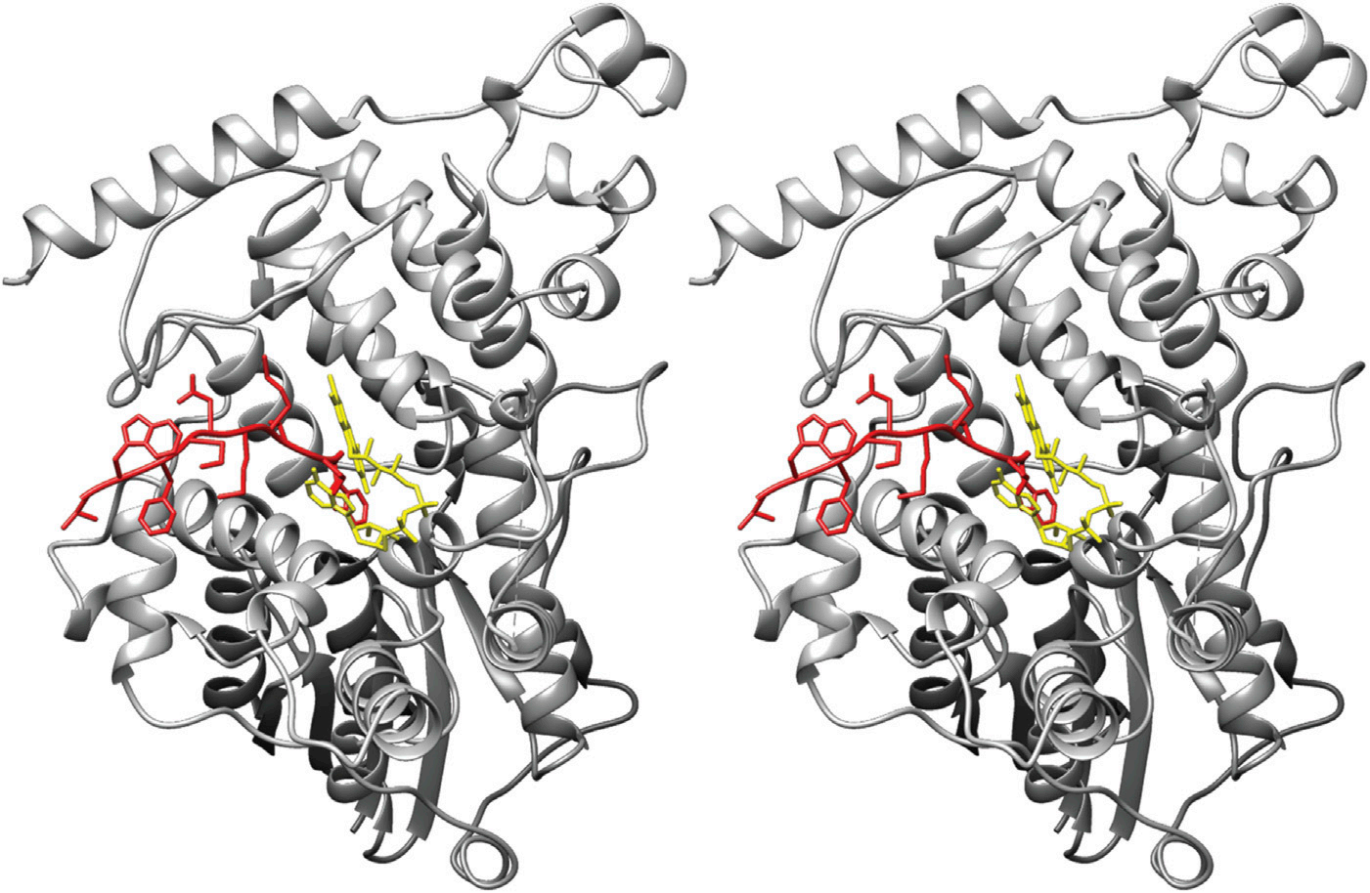
A stereoview of murine cryptochrome 2 with bound FAD (yellow) (PDB ID code 4I6G). Ten C-terminal amino acids of the ubiquitin ligase Fbxl3 were superimposed from the Cry2-Fbxl3-Skp1 complex structure (PDB ID code 4I6J) and are colored in red.

A remarkable molecular configuration was unveiled by the atomic structure of the complex between the nearly full-length version of murine Cry2 and the Fbxl3-Skp1 dimer (Xing et al., 2013). It turned out that the last residue of the Fbxl3 tail, tryptophan 428, reaches the core of the FAD-binding pocket and occupies the cofactor site. To enter the pocket, the Fbxl3 tail stacks its P426 against W310 of Cry2 and additionally flips H373 and T427 of Cry2. In the FAD-bound state, these residues directly interact with the cofactor (Xing et al., 2013). The structure suggested that FAD and Fbxl3 compete for binding to cryptochrome (Figure 1). Indeed, applying FAD *in vitro*, the authors were able to release Fbxl3 from the complex. In the case of the murine Cry1, there are no structural data regarding its interaction with Fbxl3. Nevertheless, careful analyses of the crystal structure of the unliganded Cry1 suggested that association of Fbxl3 across the FAD-binding pocket was possible as well (Czarna et al., 2013).

It remains to be proven that the paralogue Fbxl21 engages in a similar interaction with the FAD-binding pocket of cryptochromes. The C-terminal tails of the human and murine Fbxl21 have proline and tryptophan at the identical positions as in Fbxl3, the structural features that would allow Fbxl21to compete with FAD for the cofactor-binding pocket. Of note, the overall strength of the interaction between Fbxl21 and Cry was reported to be higher when compared to the Fbxl3-Cry interaction according to pulldown experiments (Yoo et al., 2013). Different reasons might underlie the increased binding affinity in cellular extracts, such as additional interactors and posttranslational modifications. More structural data is required to understand the Cry-Fbxl21 interaction and its dependence on FAD.

In contrast to Fbxl3 binding, the interaction of murine Cry1 and Per2 was found to leave the FAD-binding pocket accessible and unoccupied (Schmalen et al., 2014). Detailed inspection of the conformation of individual residues in the pocket allowed the authors to propose a slightly negative effect of Per2 association on the binding of FAD. Yet the presence or absence of FAD in the complex could not be inferred unambiguously based on the available structural evidence alone. On the other hand, the data allowed the authors to conclude that the Per2 and Fbxl3 association with Cry1 is mutually exclusive, which suggests that Per2 stabilizes the cryptochrome by shielding it from ubiquitylation (Schmalen et al., 2014).

## 8 FAD pocket-binding compounds protect cryptochromes from degradation

The docking of ubiquitin ligase SCF^Fbxl3^ onto the FAD-binding pocket of cryptochromes suggests a way to protect them from ubiquitylation by small molecule competitors targeted to that protein site. Actually, the proof of this principle had been published even before the structure of Cry2-Fbxl3-Skp1 was solved. Specifically, workers screened a library of ca. 60000 compounds for effects on Bma1l-dLuc reporter and identified three carbazole derivatives that lengthened the period of luminescence rhythms (Hirota et al., 2012). One of them, KL001, was used to prepare an affinity agarose that enabled purification and identification of its binders Cry1, Cry2 and to a lower extent Per1. FAD cofactor, when used at low millimolar concentrations, inhibited Cry1 interaction with the affinity resin. KL001 inhibited ubiquitylation of Cry1 *in vitro* indicating an impaired interaction of cryptochrome with the ubiquitin ligase Fbxl3.

**FIGURE 2:**
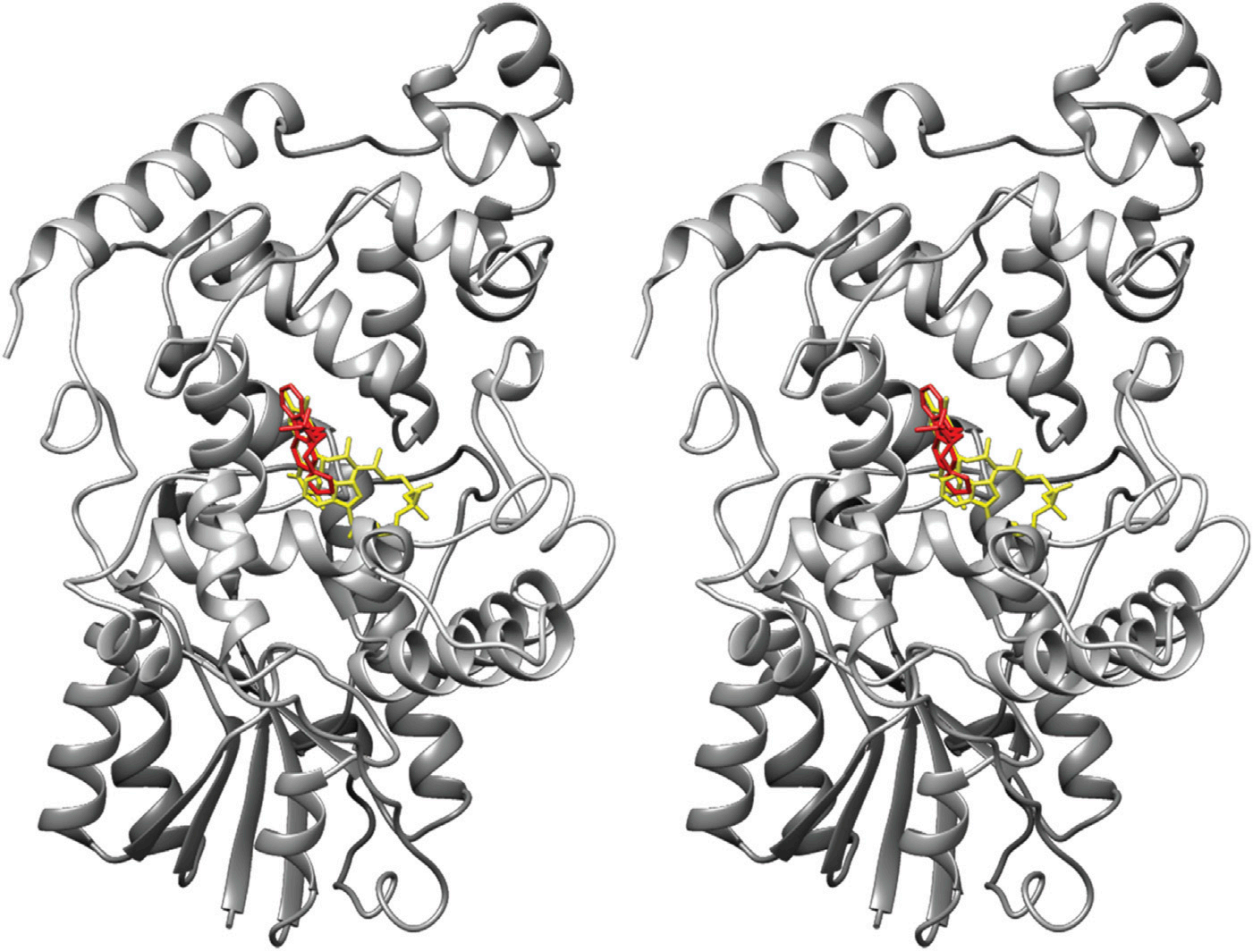
A stereoview of murine cryptochrome 2 with bound KL001 (red) (PDB ID code 4MLP). The position of FAD (yellow) in the FAD-binding pocket was superimposed from the Cry2-FAD structure (PDB ID code 4I6G).

Since FAD was able to compete with both Fbxl3 and KL001, it seemed plausible that KL001 docks onto the FAD-binding pocket of cryptochromes. This assumption was confirmed when the crystal structure of Cry2 in complex with KL001 was solved (Nangle et al., 2013). The structure revealed the chimeric nature of KL001: its carbazole ring bound similarly to FAD, the other half imitated structurally the tail of the ubiquitin ligase Fbxl3 (Figure 2). The authors observed a noteworthy structural sensitivity of the phosphate-binding loop in the FAD-binding pocket to a tyrosine residue of a neighbour protein in the crystal. This sensitivity together with the loop sequence conservation prompted the authors to suggest a possible role of protein-protein interactions in modulating the affinity of Cry to FAD (Nangle et al., 2013).

Meanwhile, several other small molecules have been developed and characterized, which bind to the FAD pocket and act in an isoform-nonselective and isoform-selective manner. In addition to KL001-like activators, small molecule inhibitors of cryptochromes have been established as well, such as KS15 (Chun et al., 2014), however, there are no structural data regarding their mode of association with cryptochromes yet. In contrast, binding of a number of activators has been successfully analysed by means of X-ray crystallography. The isoform selectivity of some of them, such as KL101, KL201, and TH301 (Miller et al., 2020b; Miller et al., 2020a), is remarkable given the high sequence and structural similarity of the FAD-binding pocket in the isoforms of cryptochromes. A partial explanation of this selectivity is the requirement of the C-terminal tail for the interaction (Miller et al., 2020b). As opposed to the similarity of the pockets, the disordered C-terminal tails of cryptochromes show high sequence divergence, which might be the structural basis for isoform-specific effects of the activators. Indeed, swapping of the cryptochrome tails abolished the binding of KL101 and TH301 to Cry1 and Cry2, respectively. An additional determinant of the isoform-specificity of the activator compounds was identified in a recent study that described conformational isomerism of the gatekeeper tryptophan in the FAD-binding pockets of Cry1 and Cry2 (Miller et al., 2021). As expected, the isoform-nonselective compound KL001 could accommodate with both conformations of the gatekeeper tryptophan, the Cry1- and Cry2-specific, without apparent steric clashes.

## 9 FAD supplementation increases intracellular levels of cryptochromes

An early indication of the *in vivo* importance of FAD for cryptochrome function came from the overexpression of *Potorous tridactylus* photolyase in Cry1/Cry2-double knockout mice (Chaves et al., 2011a). The photolyase could act as a true cryptochrome and restored the molecular oscillator in the liver of these mice. Recently, it was shown that a missense mutation (Ala260 to Thr) is responsible for familial advanced sleep phase (FASP), a heritable human sleep disorder (Hirano et al., 2016). The Ala260 resides in the FAD binding pocket, which suggested that FAD binding might modulate cryptochrome function *in vivo*. Indeed, FAD supplementation of cell culture medium increased steady-state levels of transfected Cry1 and Cry2 in 293T cells (Hirano et al., 2017). Similarly, endogenous cryptochromes could be stabilized with additional FAD in NIH 3T3 cells, albeit more weakly, when measured in a cycloheximide chase assay which quantifies protein degradation rates *in vivo*. Very high concentrations of FAD were required in those experiments, which might be due to the fact that the added FAD must be converted to riboflavin first to be taken up by cells. As mentioned earlier, intracellular riboflavin is then phosphorylated back to FMN by riboflavin kinase (Rfk) followed by adenylation of FMN to FAD by flavin adenine dinucleotide synthetase 1 (Flad1). In support of a role for FAD in cryptochrome stability, transient and stable knockdowns of Rfk in NIH 3T3 cells reduced the steady state levels and stability of transfected and endogenous cryptochromes (Hirano et al., 2017). The authors went on to verify these effects using riboflavin-free diet and siRNA against Rfk in mice. Interestingly, a circadian fluctuation of nuclear FAD levels in mouse liver was detected, which is in line with the role of the cofactor in fostering cryptochromes structurally and functionally. The fluctuation was observed under a light-dark cycle and the mechanism of its generation remains to be determined.

## 10 Conclusion

Over the last years, many new structural and functional details of animal cytochromes have been uncovered, which allows us to appreciate the complexity and beauty of the molecular clocks that drive our circadian cycles. However, the question regarding the role of the cofactor FAD in Type II cryptochromes remains open. Currently, there is not much direct evidence that FAD is necessary for the function of mammalian cryptochromes. The available data is mostly from *in vitro* reconstitutions and *in vivo* experiments where rather high levels of flavins were applied. The structural data, including the recent analyses of the cryptochrome-binding small molecules, suggest a competition between FAD and Cry interactors for association with cryptochromes, yet the biological role of the competition model has yet to be verified.

Given the weak binding, more biophysical observations in cells and in relevant subcellular compartments might be helpful. Biochemically, the identification of new partner proteins and analyses of cryptochromes together with their interactors would help clarify the circumstances and importance of FAD binding. Finally, experimental models allowing manipulation of flavin household globally or tissue-specifically would likely uncover new links between cryptochrome function, metabolism and the circadian physiology.

## Abbreviations

CCT, C-terminal extension of cryptochromes; Cry, cryptochrome; ENU, N-ethyl-N-nitrosurea; FAD, flavin adenine dinucleotide; Flad1, flavin adenine dinucleotide synthetase 1; FMN, flavin mononucleotide; Per, period; PHR, photolyase homology region; Rfk, riboflavin kinase.
